# Self‐Reported Adverse Events Following COVID‐19 Vaccination Among Medical Sciences Students After a Symptomatology Training Program: A Cross‐Sectional Study

**DOI:** 10.1002/hsr2.70492

**Published:** 2025-03-02

**Authors:** Taraneh Tavanaei Tamanaei, Mohammad Bagher Oghazian, Mehran Mojtabaee, Mojdeh Faregh, Sahar Oghazian, Erfan Tavana, Amin Hoseinzadeh, Ramin Haghighi

**Affiliations:** ^1^ Lung Diseases Research Center Mashhad University of Medical Sciences Mashhad Iran; ^2^ Clinical Research Development Unit, Imam Hassan Hospital North Khorasan University of Medical Sciences Bojnurd Iran; ^3^ Department of Internal Medicine, Faculty of Medicine North Khorasan University of Medical Sciences Bojnurd Iran; ^4^ Student Research Committee North Khorasan University of Medical Sciences Bojnurd Iran; ^5^ Department of Urology, Faculty of Medicine North Khorasan University of Medical Sciences Bojnurd Iran

**Keywords:** adverse events, AZD1222, BBIBP‐CorV, bias, COVID‐19, vaccination

## Abstract

**Background and Aims:**

Accurate and transparent investigation of adverse events (AEs) following COVID‐19 vaccination enhances social trust and confidence in vaccination programs. This study aimed to assess the prevalence of COVID‐19 vaccination‐related AEs among medical sciences students.

**Methods:**

In this cross‐sectional study, a self‐administered survey via Google Forms was conducted to evaluate AEs associated with the AZD1222 (Oxford‐AstraZeneca) and BBIBP‐CorV (Sinopharm) vaccines among medical sciences students. Data were collected following participation in a training program focused on the symptomatology of AEs, which was designed to reduce confirmation bias.

**Results:**

A total of 263 medical sciences students from North Khorasan University of Medical Sciences, Bojnurd, Iran, participated in this study, with data collection occurring from August 8 to September 1, 2021. The median age of the study population was 23 years (IQR: 22–24), with 207 participants being female (78.7%). Following the first dose, the prevalence of AEs was significantly more common in the Oxford‐AstraZeneca group compared to the Sinopharm group [OR 12.93 (5.57–30.03), *p* < 0.001]. However, in the second dose, there was no significant difference in the prevalence of AEs between the Oxford‐AstraZeneca group compared to the Sinopharm group [OR 1.45 (0.86–2.46), *p* = 0.17]. Injection site pain, fever, body/muscle pain, headache, feeling unwell, and fatigue were the most common AEs associated with both vaccines, with variations in the prevalence between the vaccines and between doses. The type of vaccine and sex were the only factors influencing the prevalence of AEs. Notably, the odds of experiencing AEs were higher in women compared to men.

**Conclusions:**

The findings of this study indicated that the prevalence of AEs was higher for the Oxford‐AstraZeneca vaccine compared to the Sinopharm vaccine. Further research is necessary to explore the impact of utilizing standardized definitions and common terminology for COVID‐19 vaccination‐related AEs in ensuring accurate and consistent reporting.

## 1 Background

The novel coronavirus disease 2019 (COVID‐19) was identified at the end of December 2019 [1] and rapidly spread across the globe, leading to the declaration of a worldwide pandemic on March 11, 2020 [2]. Vaccines were swiftly developed to achieve herd immunity [3, 4]. Sputnik V by the Gamaleya Research Institute, Russia, was the first vaccine for COVID‐19 to be registered in the world, but its safety and efficacy required further verification [5]. The World Health Organization (WHO) granted the first emergency use authorization (EUA) for a COVID‐19 vaccine to Pfizer‐BioNTech (BNT162b2) on December 31, 2020, to facilitate its global production and distribution [6]. On December 2, 2020, the United Kingdom became the first country to authorize the emergency use of BNT162b2 [7].

The other vaccines were produced gradually and received national and international approvals over time [8]. According to the latest update of the COVID‐19 vaccine tracker and landscape published on March 30, 2023, 183 candidate vaccines on 11 platforms are in clinical development to struggle with COVID‐19 [9]. Among them, only 12 vaccines have granted EUA for COVID‐19, including the Oxford‐AstraZeneca and Sinopharm vaccines [10].

The Jenner Institute at the University of Oxford and the British‐Swedish pharmaceutical company AstraZeneca collaborated to develop a non‐replicating chimpanzee adenovirus vector vaccine, formerly known as ChAdOx1nCoV‐19 and now called AZD1222. This vaccine encodes the complete S protein of SARS‐CoV‐2, which stimulates the immune system for the real exposure of SARS‐CoV‐2 [4, 8]. AZD1222 received its first EUA from the United Kingdom on December 30, 2020, and the WHO approved it for emergency use on February 15, 2021 [4, 8]. The recommended dosage is two separate doses administered intramuscularly with an interval of 8–12 weeks [11]. Several studies have reviewed and evaluated the efficacy of this vaccine [12, 13, 14, 15]. Among them, one systematic review reported a 78% protection against hospitalization and a ~63% reduction in the number of positive cases detected among vaccinated individuals [14]. The efficacy of the AZD1222 vaccine is lower than that of the mRNA vaccines [13, 15]. The AZD1222 vaccine, a viral vector vaccine, is among the vaccines that have a high incidence of adverse events (AEs) [13, 16, 17]. Vascular complications, such as thrombosis, have increased with the use of this vaccine, raising serious concerns, although it is rare [15, 17, 18].

The Sinopharm COVID‐19 vaccine, also known as BBIBP‐CorV or BIBP vaccine, was developed by the Beijing Institute of Biological Products (BIBP), sometimes written as Beijing Bio‐Institute of Biological Products (BBIBP), a subsidiary of the China National Pharmaceutical Group Corporation (Sinopharm). The vaccine received approval for general use in China and the United Arab Emirates in December 2020 and later obtained EUA from the WHO in May 2021 [19]. The Sinopharm vaccine is an inactivated whole‐virus vaccine that contains aluminum‐hydroxide‐adjuvant [20]. The recommended interval between the two doses is 3–4 weeks [21]. The Sinopharm vaccine has been reported to have an efficacy of 69% [22] and 79.3% [13] in two different studies. A recent comprehensive analysis of the efficacy and effectiveness of inactivated COVID‐19 vaccines estimated a pooled efficacy of 71.9% for inactivated vaccines, which includes the Sinopharm vaccine [23]. Multiple studies have reported that inactivated vaccines have the lowest incidence of AEs when compared to mRNA and viral vector vaccine platforms [24, 25, 26].

The short interval between the pandemic and the production and distribution of the vaccines raised concerns about their efficacy and immunogenicity [4]. Safety assessments in the preliminary stages relied solely on the reports of the initial phases of the primary clinical trials conducted by the vaccine manufacturers. However, post‐authorization safety surveillance has consistently revealed that the AEs related to COVID‐19 vaccines are predominantly mild to moderate in severity [17, 26, 27, 28].

Pharmacovigilance methods are classified into three types: active, passive, and hybrid [29]. Active surveillance involves collecting data on AEs from self‐report instruments, which may be subject to unreliability or self‐reporting bias [30]. One strategy to overcome confirmation bias in cross‐sectional observational studies utilizing self‐reporting instruments is to implement training and education programs [30]. Additionally, to confirm the accuracy of measurements, it is crucial to provide participants with standardized definitions of symptoms [30, 31, 32]. Consequently, participants in our study completed a symptomatology training program that offered clear and consistent definitions of symptoms before self‐reporting any AEs. The aim of this study was to evaluate the AEs of AZD1222 (Oxford‐AstraZeneca) and BBIBP‐CorV (Sinopharm) vaccines among medical students of North Khorasan University of Medical Sciences who had completed this program before participating.

## 2 Methods

### 2.1 Population

A cross‐sectional study was conducted among medical sciences students to assess AEs following COVID‐19 vaccination using a self‐administered Google Forms survey. Participants were medical sciences students enrolled in clinical courses during their academic program, all of whom had voluntarily received two doses of COVID‐19 vaccines. The study population included students from various disciplines, such as general medicine, midwifery, dentistry, nursing, laboratory sciences, and radiologic technology, who were available for the census method.

### 2.2 Participation

The Office of Education and Instructional Services (OEIS) of the university faculties provided data on students who had received two doses of the vaccine and were enrolled in clinical courses. This data included the students' names, fields of study, and contact information.

Initial information regarding participation in this research was disseminated to the students via short messages on mobile phones facilitated by the OEIS. The following day, more comprehensive information was sent via email, emphasizing the significance of participating in the study on vaccine AEs. The email requested that participants read and sign the consent form within 1 week if they wished to participate in the study and attend the symptomatology training program before the commencement of the study.

### 2.3 Symptomatology Training Program

All students participating in the study had completed the medical symptomatology course as part of their academic program. However, the study researchers believed that reviewing the definitions of symptoms included in the study questionnaire during a training program could facilitate more efficient, accurate, and timely completion of the form by the students. Therefore, a clinical pharmacist conducted a 2‐h session to train the participants on the symptomatology of AEs and the completion of the questionnaire. The training program successfully completed the training of all volunteers within 1 week.

The clinical pharmacist ensured accurate and consistent descriptions of AEs symptomatology by using the brief definition provided for each AE in the Common Terminology Criteria for Adverse Events (CTCAE) version 5.0 [33]. When necessary, he also referred to the Description section of TheFreeDictionary 2020 by Farlex Inc. within the Medical Dictionary subsection to further clarify the meaning of each AE.

### 2.4 Instrument

In this study, we utilized a questionnaire whose validity and reliability had been previously assessed by our team [34]. Subsequently, the questionnaire was adapted into a Google Form for data collection.

### 2.5 Study Implementation

The study participants received both doses of the available vaccines, including Oxford‐AstraZeneca (AZD1222 or ChAdOx1 nCoV‐19, South Korea, viral vector) and Sinopharm (BBIBP‐CorV, China, inactivated whole virus). Participants were allowed to choose the type of vaccine they received, and the same type was administered for both doses. Each participant was authorized to complete the study form upon finishing the symptomatology training program. To prevent missing data, all fields in the form were marked as “required.” The researchers addressed participants' questions over the phone while they filled out the form.

### 2.6 Statistical Methods of Data Analysis

Discrete data were presented as frequencies and percentages, and analyzed using the Chi‐squared test. Continuous data were expressed as median (interquartile range [IQR]) or mean (standard deviation), and compared using the Mann–Whitney *U*‐test or the Independent Samples *T*‐test, as appropriate. Logistic regression analysis was employed to examine the association between vaccine type (Oxford‐AstraZeneca and Sinopharm) and the likelihood of each outcome occurring, with results displayed as odd ratios (OR) and 95% confidence intervals (CI). All tests were two‐sided, and a *p*‐value of ≤ 0.05 was considered statistically significant. Statistical analysis was performed using IBM SPSS Statistics for Windows, version 22.0 (IBM Corp., Armonk, NY).

### 2.7 Ethical Considerations

Before the study commenced, students received an email detailing the study's objectives and significance. They subsequently read and signed the consent form, which emphasized voluntary participation and the right to withdraw without consequences. Additionally, the consent form clarified that no incentives would be provided to participants. The study form was completed online using the Google Forms platform and, with no paper forms utilized. Participants received assurance that the information collected via Google Forms would remain confidential and would not be disclosed under any circumstances. This study was part of a larger research project investigating the AEs of COVID‐19 vaccines. The larger research project was supported by the North Khorasan University of Medical Sciences through grant number 4000036 and received approval from the university's ethics committee under the reference IR.NKUMS.REC.1400.062. All data collection and analysis were conducted in compliance with the principles outlined in the Declaration of Helsinki [35].

## 3 Results

### 3.1 Study Participants: Sampling Process and Key Characteristics

During the screening phase, 406 students enrolled in clinical courses were initially assessed. Among them, 105 students were ineligible for assessment as they had either received only the first vaccine dose or were awaiting vaccination. Of the remaining 301 fully vaccinated students, 38 (12.6%) opted out for personal reasons. The study ultimately included 263 students (87.4%), from whom all relevant data were collected between August 8 and September 1, 2021, and subsequently analyzed. Of these, 156 (59.3%) received the Oxford‐AstraZeneca vaccine, and 107 (40.7%) were vaccinated with Sinopharm. The participants comprised 207 females (78.7%) and 56 males (21.3%), aged 20–41 years, with a median age of 23 years (IQR: 22–24). There were no significant differences between the two vaccine groups regarding the history of COVID‐19 before or after vaccination, the frequency of contracting COVID‐19 before vaccination, underlying diseases, medication intake, or the history of receiving the influenza vaccine. In contrast, sex, age, body mass index, the interval between the first and second dose administrations, the time from the second dose to participation in the symptomatology training program, and the academic field of study showed significant differences between the study groups. The characteristics and demographic details of the study population are presented in Table 1.

**Table 1:** Characteristics and demographic details of the study population.

Characteristics	Vaccine type		*p* value
	Oxford‐AstraZeneca (*n* = 156)	Sinopharm (*n* = 107)	
Sex, *N* (%)			**< 0.001**
Female	108 (69.2%)	99 (92.5%)	
Male	48 (30.8%)	8 (7.5%)	
Age (year)			
Median (IQR)	24 (23–25)	22 (21–23)	**< 0.001** a
Minimum–maximum	21–36	20–41	
Mean (standard deviation)	24.25 (2)	22.41 (2.61)	
Body Mass Index (kg/m^2^)			
Median (IQR)	22.27 (20.58–23.92)	20.76 (19.05–22.91)	**< 0.001** a
Minimum–maximum	18–38.22	16–31.96	
Mean (standard deviation)	22.8 (3.38)	21.3 (3.13)	
Underlying diseases, *N* (%)			0.55
Yes	23 (14.7)	13 (12.1)	
No	133 (85.3)	94 (87.9)	
Medication intake, *N* (%)			0.27
Yes	15 (9.6)	15 (14)	
No	141 (90.4)	92 (86)	
Academic field of study, *N* (%)			**< 0.001**
General medicine	121 (77.6)	31 (29.0)	
Midwifery	14 (9.0)	35 (32.7)	
Dentistry	19 (12.2)	1 (0.9)	
Nursing	0	15 (14.0)	
The total of radiologic technology and laboratory sciences	2 (1.3)	25 (23.4)	
The interval between the first and second dose administrations, median (IQR), days	93 (93–93)	31 (30–31)	**< 0.001** a
Time from the second dose to participation in the symptomatology training program, median (IQR), days	25 (21–28)	29 (11–50)	**0.04** a
History of COVID‐19 before vaccination, *N* (%)			0.17
Yes	70 (44.9)	39 (36.4)	
No	86 (55.1)	68 (63.6)	
Frequency of contracting COVID‐19 before vaccination, *N* (%)			0.29
1	49 (70)	32 (82.1)	
2	19 (27.1)	7 (17.9)	
3	2 (2.9)	0	
History of receiving the influenza vaccine, *N* (%)			0.12
Yes	32 (20.5)	14 (13.1)	
No	124 (79.5)	93 (86.9)	
COVID‐19 after vaccination, *N* (%)	20 (12.8)	11 (10.3)	0.53

*Note:* Bold formatting is utilized to denote statistically significant values.

Abbreviation: IQR, interquartile range.

^a^ Nonparametric Mann–Whitney test.

### 3.2 Details of Other Characteristics

Among the underlying diseases, our findings indicated that allergic reactions were the most common, affecting 36.1% (*n* = 13) of participants with pre‐existing diseases (*n* = 36). Furthermore, there were no reported cases of hypertension, joint disorders, liver diseases, ophthalmological conditions, chronic obstructive pulmonary disease, diabetes, or cancer.

The evaluation of vaccine confidence revealed significantly greater trust in the Oxford‐AstraZeneca vaccine compared to the Sinopharm vaccine across both doses (*p* < 0.001). Notably, the prevalence of fear associated with the first dose of Oxford‐AstraZeneca was significantly higher than that reported with Sinopharm (34.6% vs. 13.1%, *p* < 0.001). However, regarding the second dose, there was no significant difference in vaccine‐related fear between the two groups (*p* = 0.24). Furthermore, the reduction in the prevalence of vaccine‐related fear from the first dose to the second dose was more pronounced in the Oxford‐AstraZeneca group than in the Sinopharm group.

### 3.3 AEs Following the First and Second Doses

As shown in Table 2, the prevalence of reported AEs was significantly higher in the Oxford‐AstraZeneca group compared to the Sinopharm group following the first dose [OR 12.93 (5.57–30.03), *p* < 0.001]. However, no significant difference was observed between the two groups regarding the prevalence of AEs after the second dose [OR 1.45 (0.86–2.46), *p* = 0.17]. Despite the prevalence of AEs being significantly higher after the first dose compared to the second dose in Oxford‐AstraZeneca recipients [OR 9.17 (4.60–18.30), *p* < 0.001], there was no significant difference in the prevalence of AEs between the first and second doses in Sinopharm recipients (*p* = 0.58). Additionally, the number of AEs per participant was significantly higher in the Oxford‐AstraZeneca group compared to the Sinopharm group for both doses (*p* < 0.001). Within the Oxford‐AstraZeneca group, the number of AEs per participant was significantly higher after the first dose compared to the second dose [OR 1.93 (1.58–2.37), *p* < 0.001]. In contrast, the Sinopharm group did not show a significant difference between the first and second doses in this regard (*p* = 0.37). A detailed distribution of AEs prevalence is provided in Table 2.

**Table 2:** Distribution of adverse events prevalence, recipients' perspectives, symptom‐alleviating medications, and vaccine outcomes.

Characteristics		Vaccine types		
	Total	Oxford‐AstraZeneca	Sinopharm	OR (0.95% CI, *p* value)^a^
Frequency of participants with adverse events, *N* (%)	*N* = 263	*N* = 156	*N* = 107	
First dose	208 (79.1%)	145 (92.9%)	63 (58.9%)	**12.93 (5.57–30.03, < 0.001)** b
Second dose	151 (57.4%)	92 (59%)	59 (55.1%)	1.45 (0.86–2.46, 0.17)^b^
OR (0.95% CI, *p*‐value) *between* doses^c^	**2.81 (1.91–4.12, < 0.001)**	**9.17 (4.60–18.30, < 0.001)**	1.16 (0.68–2.00, 0.58)	
One of both doses	232 (88.2)	149 (95.5)	83 (77.6)	**9.56 (3.43–26.65, < 0.001)** b
Both doses	127 (48.3)	88 (56.4)	39 (36.4)	**2.85 (1.66–4.89, < 0.001)** b
Number of adverse events, median (IQR)	*N* = 263	*N* = 156	*N* = 107	
First dose	3 (2–5)	4 (3–6)	1 (1–2)	**2.80 (2.16–3.62, < 0.001)**
Second dose	1 (1–2)	2 (1–3)	1 (1–2)	**1.73 (1.19–2.51, 0.004)**
OR (0.95% CI, *p*‐value) *between* doses^d^	**1.71 (1.45–2.00, < 0.001)**	**1.93 (1.58–2.37, < 0.001)**	1.24 (0.77–2.00, 0.37)	
The perspective of vaccine recipients on the severity, duration, and number of adverse events among those who experienced adverse events with both doses				
Severity	*N* = 127	*N* = 88	*N* = 39	
The first dose is more than the second	97 (76.4)	77 (87.5)	20 (51.3)	**8.80 (3.18–24.29, < 0.001)**
The second dose is more than the first	23 (18.1)	7 (8)	16 (41)	Reference
Similar	7 (5.5)	4 (4.5)	3 (7.7)	3.05 (0.53–17.37, 0.21)
Duration	*N* = 127	*N* = 88	*N* = 39	
The first dose is more than the second	96 (75.6)	76 (86.4)	20 (51.3)	**7.60 (2.85–20.28, < 0.001)**
The second dose is more than the first	24 (18.9)	8 (9.1)	16 (41)	Reference
Similar	7 (5.5)	4 (4.5)	3 (7.7)	2.67 (0.48–14.90, 0.26)
Number	*N* = 127	*N* = 88	*N* = 39	
The first dose is more than the second	82 (64.6)	71 (80.7)	11 (28.2)	**8.30 (2.56–26.85, < 0.001)**
The second dose is more than first	16 (12.6)	7 (8)	9 (23.1)	Reference
Similar	29 (22.8)	10 (11.4)	19 (48.7)	0.68 (0.19–2.36, 0.54)
Participants who used medication(s) to alleviate symptoms, *N* (%)	*N* = 263	*N* = 156	*N* = 107	
First dose	158 (60.1)	130 (83.3)	28 (26.2)	**14.11 (7.72–25.77, < 0.001)**
Second dose	69 (26.2)	57 (36.5)	12 (11.2)	**4.56 (2.30–9.03, < 0.001)**
Days of medication(s) use, median (IQR)^e^				
First dose	1 (1–2)	1 (1–2)	1 (1–1)	1.04 (0.63–1.73, 0.86)
Second dose	1 (1–1)	1 (1–1)	1 (1–2)	0.76 (0.46–1.27, 0.30)
Improvement of symptoms, *N* (%)^e^				
First dose	*N* = 158	*N* = 130	*N* = 28	1.11 (0.29–4.28, 0.88)
< 4 h	122 (77.2)	100 (76.9)	22 (78.6)	
> 4 h	36 (37.2)	30 (23.1)	6 (21.4)	
Second dose	*N* = 69	*N* = 57	*N* = 12	0.21 (0.03–1.61, 0.13)
< 4 h	61 (88.4)	52 (91.2)	9 (75)	
> 4 h	8 (11.6)	5 (8.8)	3 (25)	
Hospitalization following the adverse events, *N* (%)	*N* = 263	*N* = 156	*N* = 107	
First dose	7 (2.7)	6 (3.8)	1 (0.9)	4.24 (0.50–35.73, 0.18)
Leaving academic program following vaccine administration, *N* (%)	*N* = 263	*N* = 156	*N* = 107	
First dose	112 (42.6)	107 (68.6)	5 (4.7)	**44.55 (17.07–116.26, < 0.001)**
Second dose	21 (8.0)	18 (11.5)	3 (2.8)	**4.52 (1.30–15.76, 0.02)**

*Note:* Bold formatting is utilized to denote statistically significant values.

^a^ Odds ratio (95% confidence interval). The Sinopharm group was designated as the reference group, serving as the baseline for comparison.

^b^ Adjusted for sex.

^c^ The reference category is the second dose.

^d^ The reference category is the second dose. An example for interpretation is as follows: “The odds of experiencing any additional adverse event are 1.93 times higher with the first dose of the Oxford‐AstraZeneca vaccine compared to its second dose.”

^e^ Among those participants had adverse events and received medications to relieve symptoms.

As mentioned in the Supporting Information S1: Table S1, the most frequent AEs following the first dose of Oxford‐AstraZeneca and Sinopharm were fever (*n* = 113, 72.4%) and injection site pain (*n* = 39, 36.4%), respectively. However, injection site pain was the most frequent AE following the second dose of Oxford‐AstraZeneca (*n* = 58, 37.2%) and Sinopharm (*n* = 40, 37.4%) with a nonsignificant between‐group difference [OR = 0.99 (0.60–1.65), *p* = 0.97]. According to the investigation of AEs odds following the first dose, participants who received Oxford‐AstraZeneca were significantly more prone to report a higher frequency of injection site pain, fever, body/muscle pain, chills, headache, feeling unwell, fatigue, sweating, nausea, and dizziness compared to those who received the Sinopharm vaccine (Supporting Information S1: Table S1). Consistently, the Oxford‐AstraZeneca group experienced fever, body/muscle pain, and fatigue more frequently than the Sinopharm group following the administration of the second dose. Detailed information, including odds ratios, on the prevalence of all AEs following the first and the second doses of the vaccines is provided in Supporting Information S1: Table S1.

Certain AEs were either uncommon or absent among the vaccine recipients, as detailed in Supporting Information S1: Table S1. Notably, among skin AEs, only rash was reported by Sinopharm recipients, occurring solely after the second dose (*n* = 1, 0.9%). In recipients of the Oxford‐AstraZeneca vaccine, urticaria (*n* = 1, 0.6%), rash (*n* = 2, 1.3%), and itching (*n* = 2, 1.3%) were observed exclusively after the first dose. Regarding oral AEs in Sinopharm recipients, only aphthous stomatitis was reported, occurring after both doses (*n* = 1, 0.9%). Conversely, xerostomia (*n* = 1, 0.6%) and tingling of the mouth, tongue, or lips (*n* = 1, 0.6%) were the only oral AEs reported by Oxford‐AstraZeneca recipients, and these occurred solely after the first dose.

### 3.4 Comparison of Onset and Duration of AEs

The AEs following the first and second doses were analyzed for onset and duration, and compared between the two groups. As shown in Supporting Information S2: Table S2, in the Oxford‐AstraZeneca group, the symptoms with the fastest onset were tingling of the mouth/tongue/lips and local redness at the injection site. Conversely, the symptoms with the slowest onset were rash and depression. In the Sinopharm group, the symptom with the fastest onset was local stiffness, while the symptom with the slowest onset was rhinorrhea. Regarding symptom duration in the Oxford‐AstraZeneca group, hypotension and local warming had the shortest durations, whereas depression was associated with the longest duration. In the Sinopharm group, the symptoms with the shortest durations were nausea, chills, and sweating, while aphthous stomatitis and hypotension exhibited the longest durations.

When comparing the onset of symptoms following the first dose, the median (IQR) onset times for injection site pain, body/muscle pain, headache, feeling unwell, fatigue, and local stiffness were significantly shorter in the Sinopharm group than in the Oxford‐AstraZeneca group. Consequently, these AEs appeared earlier in the Sinopharm group. The comparison of symptom duration between the two groups revealed a significant difference only in the duration of injection site pain, which was significantly shorter in the Sinopharm group compared to the Oxford‐AstraZeneca group.

Supporting Information S3: Table S3 presents the time to onset and duration of AEs following the second dose of vaccination. In the Oxford‐AstraZeneca group, the fastest onsets were observed for injection site pain, body/muscle pain, local stiffness, and sweating, whereas the slowest onset was noted for local warming. For Sinopharm recipients, the fastest onsets were for injection site pain and dizziness, with the slowest onset observed for rash. Regarding symptom duration in the Oxford‐AstraZeneca group, chills exhibited the shortest duration, while local stiffness persisted the longest. In the Sinopharm group, local swelling and local warming had the shortest durations, whereas rash and aphthous stomatitis were the most prolonged.

When comparing the onset and duration of AEs between the two groups, the median (IQR) onset times for injection site pain, fever, headache, and feeling unwell were significantly earlier in the Sinopharm group compared to the Oxford‐AstraZeneca group. However, no significant differences were observed in the duration of each AE between the two groups. Supporting Information S2: Table S2 and Supporting Information S3: Table S3 provide the central tendencies and dispersion indicators of reported AEs for both the first and second doses, facilitating a comparison between the two groups.

### 3.5 The Perspective of Vaccine Recipients Regarding Severity, Duration, and Number of AEs

This study investigated the severity, duration, and number of AEs from the perspective of vaccine recipients who experienced AEs with both doses (*n* = 127). The survey results indicated that recipients of the Oxford‐AstraZeneca vaccine, compared to recipients of the Sinopharm vaccine, demonstrated significantly higher odds of experiencing greater severity, longer duration, and a higher number of AEs following the first dose relative to the second dose (Table 2).

### 3.6 Vaccine Outcomes

Among the total participants (*n* = 263), 60.1% (*n* = 158) used at least one medication to alleviate AEs following the first dose. This proportion decreased to 26.2% (*n* = 69) after the second dose. Acetaminophen, followed by nonsteroidal anti‐inflammatory drugs (NSAIDs), were the most commonly used medications for symptom relief (Supporting Information S4: Table S4). As illustrated in Table 2, participants who received the first dose of Oxford‐AstraZeneca were 14.11 times more likely to use medication(s) to relieve symptoms compared to those who received the first dose of Sinopharm (95% CI = 7.72–25.77, *p* < 0.001). Following the second dose, participants who received Oxford‐AstraZeneca were 4.56 times more likely to use medication(s) to relieve symptoms compared to those who received Sinopharm (95% CI = 2.30–9.03, *p* < 0.001). However, examinations of symptom improvement in participants who used medication to alleviate symptoms showed no significant differences between the Sinopharm and Oxford‐AstraZeneca vaccines in the proportion of participants whose symptoms improved within less than 4 h or more than 4 h, for both the first and second doses (Table 2).

Hospitalization following vaccine administration occurred only after the first dose. Specifically, seven students (3.4%) reported being hospitalized for 1 day or less due to AEs. Of these, six students had received the Oxford‐AstraZeneca vaccine. Despite these hospitalizations, no students refused the second dose. However, 112 participants (42.6%) and 21 participants (8.0%) were unable to attend their academic programs and had to leave their classes following the first and second doses, respectively. Further analysis revealed that students who received the Oxford‐AstraZeneca vaccine were significantly more likely to leave their academic programs after both doses compared to those who received the Sinopharm vaccine (Table 2).

### 3.7 Factors Associated With the Prevalence of AEs

The results of multiple logistic regression models indicated that, in addition to the type of vaccine administered, sex was the only other factor influencing the prevalence of AEs among the study participants. Notably, the odds of experiencing AEs following the first dose, second dose, both doses, and at least one dose were 2.8, 2.4, 2.5, and 3.6 times higher in women compared to men, respectively. Conversely, no significant associations were found between the prevalence of AEs and other factors, particularly age, body mass index, history of COVID‐19, or underlying diseases.

## 4 Discussion

In this study, 263 medical students who completed the symptomatology training program for AEs reported the AEs associated with the Oxford‐AstraZeneca and Sinopharm vaccines via a self‐administered Google Forms survey. Among Oxford‐AstraZeneca recipients (*n* = 156), 95.5% (*n* = 149) reported at least one AE, whereas 77.6% (*n* = 83) of Sinopharm recipients (*n* = 107) reported an AE. Although the prevalence and number of AEs per participant following the first dose were higher for both vaccines compared to the second dose, this difference was statistically significant only among Oxford‐AstraZeneca recipients. The most common AEs following the first dose of Oxford‐AstraZeneca and Sinopharm were fever (*n* = 113, 72.4%) and injection site pain (*n* = 39, 36.4%), respectively. However, injection site pain was the most common AE following the second dose for both the Oxford‐AstraZeneca (*n* = 58, 37.2%) and Sinopharm (*n* = 40, 37.4%) vaccines. Skin and oral complications were uncommon among the vaccine recipients. When comparing the onset of symptoms, Sinopharm recipients experienced a faster onset for both doses compared to Oxford‐AstraZeneca recipients. Additionally, Oxford‐AstraZeneca recipients experienced a longer duration of symptoms than Sinopharm recipients for both doses, although this difference was statistically significant only for the first dose.

### 4.1 The Necessity of a Standard and Consistent Definition of AEs

A valid diagnosis of AEs requires a standard case definition [31, 32]. Utilizing a consistent case definition not only reduces the possibility of misclassification bias [36] and the underestimation or overestimation of AEs following immunization (AEFI) cases [37] during data collection but also establishes a common terminology for AEFI investigation, facilitating data comparison in clinical trials and surveillance [38]. According to the WHO, it is recommended to use the Brighton case definition if available [31, 32]. However, its application in studies related to COVID‐19 vaccines has been examined to a limited extent [37].

In the absence of a specific case definition, standard medical literature, national definitions, or other approved definitions can be utilized to support consistency in the definition of AEs [31, 32]. For this purpose, two of the most important ontologies used in pharmacovigilance are the Medical Dictionary for Regulatory Activities (MedDRA) [39] and the Systematized Nomenclature of Medicine (SNOMED) [40]. Due to limited access to specific ontologies, we could utilize the CTCAE. In the context of COVID‐19, the CTCAE has been used to grade the severity of AEs related to COVID‐19 vaccines in both cancer [41] and non‐cancer patients [42]. Additionally, it has contributed to the development of a standardized terminology and grading scale for skin AEs associated with the COVID‐19 vaccine [43]. However, our study employed the CTCAE solely to ensure consistent definitions of AEs within the symptomology training program for the participating students.

### 4.2 Oxford‐AstraZeneca

Since the initiation of COVID‐19 vaccination, numerous articles have investigated vaccine‐related AEs. Several review studies, including some with meta‐analyses, have been conducted to collect and analyze AEs reported in these articles. These reviews indicate that the prevalence of AEs is higher for viral vector vaccines (e.g., Oxford‐AstraZeneca) compared to inactivated vaccines (e.g., Sinopharm) [13, 16, 17, 24, 25, 26, 44]. Consistent with these findings, our study also demonstrates a higher prevalence of AEs for the Oxford‐AstraZeneca vaccine compared to the Sinopharm vaccine.

In a recently published study conducted via an online questionnaire among the Arab population, the prevalence of AEs following the first dose of the Oxford‐AstraZeneca and Sinopharm vaccines was 89.6% and 51.7%, respectively. After the second dose, the prevalence was 64.3% for Oxford‐AstraZeneca and 41.6% for Sinopharm [45]. Additionally, a recently published multinational randomized controlled trial (RCT) reported that the prevalence of solicited AEs for the AZD1222 vaccine was 77.7% after the first dose and 61.5% after the second dose [46]. In another cross‐sectional study conducted in northwestern Iran, Oxford‐AstraZeneca recipients who completed the questionnaire via social media or phone calls reported an AE prevalence of 84.1% after the first dose and 56.9% after the second dose [47]. However, some studies have reported a prevalence of less than 50% following the first and second doses of the Oxford‐AstraZeneca vaccine [48, 49]. It has been previously reported that AEs from the Oxford‐AstraZeneca vaccine are more common in younger individuals and females [47]. In our study, the population receiving the Oxford‐AstraZeneca vaccine was predominantly female (69.2%) and consisted mainly of students with an average age of 24 years. These factors may have contributed to the relatively high prevalence of AEs observed in our study.

In our study, the most common AEs following the first and second doses of the Oxford‐AstraZeneca vaccine were fever (72.4% and 16.7%), injection site pain (53.2% and 37.2%), body/muscle pain (52.6% and 15.4%), chills (52.6% and 8.3%), headache (40.4% and 9.0%), feeling unwell (32.7% and 10.9%), and fatigue (32.7% and 10.3%). These findings are consistent with other studies conducted in Australia and Brazil [50], Iran [51], Saudi Arabia [52], and a multinational RCT [46]. In these studies, the most common AEs of the first and second doses of the Oxford‐AstraZeneca vaccine were similar, with a higher frequency of AEs observed after the first dose compared to the second dose. However, the order of frequency of these AEs may vary between the two doses.

In another study from Iran, AEs of several vaccines, including the Oxford‐AstraZeneca vaccine, were investigated in medical students through phone calls. The results of this study showed that fever, myalgia, local site pain/swelling, weakness, and headache are the most common AEs of the Oxford‐AstraZeneca vaccine, with their frequency being higher in the first dose than in the second dose [51]. The results of a Google Forms survey‐based study on a small number of medical students in Oman showed that pain at the site of injection, generalized weakness, fever, headache, and joint pains are common AEs of the Oxford‐AstraZeneca vaccine [53]. In addition, the results of a review article showed that the prevalence of local and systemic complications in vector‐based vaccines is far more than inactivated vaccines [28]. In this regard, in a study conducted via an online questionnaire in Iran, AEs associated with the Oxford‐AstraZeneca, Sputnik V, and Sinopharm vaccines were evaluated. Common AEs reported for the first dose of the Oxford‐AstraZeneca vaccine included injection site pain, fatigue, muscle pain, fever, and chills. These AEs were more prevalent after the first dose compared to the second dose. Additionally, the study found that the prevalence of both local and systemic AEs was higher for the Oxford‐AstraZeneca vaccine than for the Sinopharm vaccine [54].

### 4.3 Sinopharm

As previously discussed, the prevalence of AEs associated with the Sinopharm vaccine is lower than that of the Oxford‐AstraZeneca vaccine. This finding has been particularly evident in studies comparing the AEs of multiple vaccines [45, 55]. In these studies, the prevalence of AEs following the first and second doses of Sinopharm was reported to range from 37.7% to 51.7%. Additionally, a multicenter study in Iran investigating the AEs of four vaccines among healthcare workers found that 93.9% of Oxford‐AstraZeneca recipients and 37.2% of Sinopharm recipients reported at least one AE following vaccination [56].

In our study, the most common AEs following the first and second doses of the Sinopharm vaccine were injection site pain (36.4% and 37.4%), feeling unwell (9.3% and 4.7%), headache (9.3% and 11.2%), fatigue (7.5% and 2.8%), fever (7.5% and 6.5%), and body/muscle pain (3.7% and 5.6%). These findings are consistent with several other studies [56, 57, 58, 59]. However, additional AEs such as chills [55, 60] and cough [60] have also been reported in some studies.

In a multi‐city prospective observational study in Iran [61], AEs during the first week following the administration of four different COVID‐19 vaccines were collected via phone calls and electronic methods. The study found that the prevalence of both local and systemic AEs was higher for the Oxford‐AstraZeneca vaccine compared to the Sinopharm vaccine. Additionally, the prevalence of these AEs was greater following the first dose than the second dose for both vaccines. The most commonly reported AE across all vaccines was injection site pain. Other AEs, which varied in frequency between the doses of Sinopharm and Oxford‐AstraZeneca vaccines, included fatigue, malaise, headache, myalgia, joint pain, and fever. Although the overall duration of AEs was not specified, the duration of both local and systemic AEs was reported to be longer for the Oxford‐AstraZeneca vaccine compared to the Sinopharm vaccine. Furthermore, while our study did not separately report the duration of local and systemic AEs, it was noted that the overall duration of symptoms was longer for the Oxford‐AstraZeneca vaccine in both doses. Also, in the mentioned study AEs did not interfere with the participants' daily activities. However, in our study, a significant number of Oxford‐AstraZeneca recipients and a smaller number of Sinopharm recipients had to leave educational programs due to AEs. The prevalence of leaving was higher following the first dose than the second dose and was significantly higher for the Oxford‐AstraZeneca vaccine compared to the Sinopharm vaccine [61].

Another study in Iran reported that 24.9% of Oxford‐AstraZeneca recipients and 3.5% of Sinopharm recipients required medical attention to alleviate symptoms [56]. Furthermore, 58.3% of Oxford‐AstraZeneca recipients and 13.5% of Sinopharm recipients were unable to perform their daily activities within 24 h postvaccination and required rest. Notably, individuals who experienced AEs following Sinopharm vaccination were significantly younger than those who did not develop AEs [56].

In another study conducted in Iran, the most common AE following Sinopharm vaccination was injection site pain, with all AEs being mild to moderate in severity. These AEs lasted less than 3 days, and no hospitalizations were reported [62]. Similar to our study, in a multicenter study conducted in Egypt, acetaminophen followed by NSAIDs were the most commonly used medications for pain or fever relief [58]. Additionally, in a study involving medical students and healthcare workers in Pakistan, where the majority of participants were female, 24.2% and 14.5% of individuals used medication to relieve symptoms after the first and second doses of the Sinopharm vaccine, respectively. These findings are consistent with our study, which reported rates of 26.2% and 11.2% [57].

### 4.4 The Association Between Various Factors and the Prevalence of AEs

In our study, females were more prone to exhibit AEs compared to males, a finding consistent with previous studies [47, 50, 61]. The observed association between sex and the prevalence of AEs can be attributed to the influence of estradiol hormone on the immune system, particularly in young females, which enhances neutralizing antibody responses and consequently results in more frequent AEs compared to males [63, 64]. Contrary to other studies [45, 47, 50, 56, 61], our research did not identify any significant association between the prevalence of AEs and other factors such as age, body mass index, history of COVID‐19, or the presence of underlying diseases.

### 4.5 Strengths and Limitations

In this study, to demonstrate the effect size, analyses were complemented with odds ratios, which is not commonly seen in studies on the prevalence of COVID‐19 vaccines, where presenting proportions is often deemed sufficient. As mentioned in the discussion section, it is crucial to adhere to the standard definition of AEs to prevent confirmation bias and, more comprehensively, misinformation bias. To this end, we provided clear and uniform definitions using the CTCAE during a symptomology training program. However, due to the cross‐sectional nature of the study, some participants may have experienced recall bias in reporting the AEs of vaccines, particularly those with a long interval between vaccine administration and study participation. Additionally, when generalizing our results, it is crucial to note that our study population consisted of medical students from a university in northeastern Iran who were predominantly female. The disparity in this demographic characteristic may have also contributed to the high prevalence of reported AEs in our study.

## 5 Conclusion

In this cross‐sectional study, accurate and consistent descriptions of AEs symptomatology were provided before self‐reporting any AEs. The findings indicated that the prevalence of AEs was higher for the Oxford‐AstraZeneca vaccine compared to the Sinopharm vaccine. Injection site pain, fever, body/muscle pain, headache, feeling unwell, and fatigue were the most common AEs associated with both vaccines, with variations in the prevalence between the vaccines and between doses. The onset of symptoms was earlier for the Sinopharm vaccine, whereas the duration of symptoms was longer for the Oxford‐AstraZeneca vaccine. following both the first and second doses, students who received the Oxford‐AstraZeneca vaccine were significantly more likely to leave their academic programs compared to those who received the Sinopharm vaccine. It is recommended that future studies with a prospective design investigate the effects of providing standard definitions and common terminology before reporting AEs in enhancing the accuracy and consistency of AE reporting for COVID‐19 vaccines.

## Author Contributions

**Taraneh Tavanaei Tamanaei:** conceptualization, methodology, validation, formal analysis, investigation, data curation, visualization, supervision, writing – original draft, writing – review and editing. **Mohammad Bagher Oghazian:** conceptualization, methodology, software, validation, formal analysis, investigation, resources, data curation, visualization, supervision, project administration, funding acquisition, writing – original draft, writing – review and editing. **Mehran Mojtabaee:** conceptualization, methodology, investigation, data curation, writing – original draft. **Mojdeh Faregh:** conceptualization, methodology, investigation, data curation, writing – original draft. **Sahar Oghazian:** conceptualization, methodology, investigation, data curation, writing – original draft. **Erfan Tavana:** methodology, investigation, visualization, writing – original draft. **Amin Hoseinzadeh:** methodology, investigation, software, writing – original draft. **Ramin Haghighi:** conceptualization, methodology, investigation, resources, writing – original draft. All authors have read and approved the final version of the manuscript. Mohammad Bagher Oghazian had full access to all of the data in this study and takes complete responsibility for the integrity of the data and the accuracy of the data analysis.

## Conflicts of Interest

The authors declare no conflicts of interest.

## Transparency Statement

The lead author, Mohammad Bagher Oghazian, affirms that this manuscript is an honest, accurate, and transparent account of the study being reported; that no important aspects of the study have been omitted; and that any discrepancies from the study as planned (and, if relevant, registered) have been explained.

## Supporting information

Supporting information.

Supporting information.

Supporting information.

Supporting information.

## Data Availability

The data that support the findings of this study are available from the corresponding author upon reasonable request.
