# Supplementary material for: Self‐Reported Adverse Events Following COVID‐19 Vaccination Among Medical Sciences Students After a Symptomatology Training Program: A Cross‐Sectional Study
Source: Health Sci Rep. 2025 Mar 2;8(3):e70492. doi: 10.1002/hsr2.70492 (PMC11872685; doi:10.1002/hsr2.70492)
Supplement: Supplementary file 1 — Supporting information. [file HSR2-8-e70492-s001.docx]

**Supplemental Table S1. Prevalence of adverse events following the first and second doses of vaccines**

| Adverse event | Vaccine types, N (%) | | | | p-value | OR  (95% CI, p-value) † |
| --- | --- | --- | --- | --- | --- | --- |
|  | Dose | Total  N = 263 | Oxford–AstraZeneca  N = 156 | Sinopharm  N= 107 |  |  |
| Injection site pain | First | 122 (46.4%) | 83 (53.2%) | 39 (36.4%) | **0.007** | **1.98 (1.20-3.28, 0.008)** |
|  | Second | 98 (37.3%) | 58 (37.2%) | 40 (37.4%) | 0.973 | 0.99 (0.60-1.65, 0.97) |
| Fever | First | 121 (46.0%) | 113 (72.4%) | 8 (7.5%) | **<0.001** | **32.52 (14.59-72.48, <0.001)** |
|  | Second | 33 (12.5%) | 26 (16.7%) | 7 (6.5%) | **0.015** | **2.86 (1.19-6.85, 0.02)** |
| Body/muscle pain | First | 86 (32.7%) | 82 (52.6%) | 4 (3.7%) | **<0.001** | **28.53 (10.01-81.30, <0.001)** |
|  | Second | 30 (11.4%) | 24 (15.4%) | 6 (5.6%) | **0.014** | **3.06 (1.21-7.77, 0.02)** |
| Chills | First | 83 (31.6%) | 82 (52.6%) | 1 (0.9%) | **<0.001** | **117.46 (15.99-862.81, <0.001)** |
|  | Second | 13 (4.9%) | 13 (8.3%) | 0 | **0.002** | >100 (0 to uncertain, >0.99) ‡ |
| Headache | First | 73 (27.8%) | 63 (40.4%) | 10 (9.3%) | **<0.001** | **6.57 (3.18-13.57, <0.001)** |
|  | Second | 26 (9.9%) | 14 (9.0%) | 12 (11.2%) | 0.550 | 0.78 (0.34-1.76, 0.55) |
| Feeling unwell | First | 61 (23.2%) | 51 (32.7%) | 10 (9.3%) | **<0.001** | **4.71 (2.26-9.79, <0.001)** |
|  | Second | 22 (8.4%) | 17 (10.9%) | 5 (4.7%) | 0.073 | 2.49 (0.89-6.98, 0.08) |
| Fatigue | First | 59 (22.4%) | 51 (32.7%) | 8 (7.5%) | **<0.001** | **6.01 (2.72-13.30, <0.001)** |
|  | Second | 19 (7.2%) | 16 (10.3%) | 3 (2.8%) | **0.022** | **3.96 (1.12-13.95, 0.03)** |
| Sweating | First | 26 (9.9%) | 25 (16.0%) | 1 (0.9%) | **<0.001** | **20.23 (2.70-151.75, 0.003)** |
|  | Second | 6 (2.3%) | 6 (3.8%) | 0 | 0.084 | >100 (0 to uncertain, >0.99) ‡ |
| Nausea | First | 16 (6.1%) | 15 (9.6%) | 1 (0.9%) | **0.004** | **11.28 (1.47-86.71, 0.020)** |
|  | Second | 4 (1.5%) | 4 (2.6%) | 0 | 0.148 | >100 (0 to uncertain, >0.99) ‡ |
| Dizziness | First | 16 (6.1%) | 14 (9.0%) | 2 (1.9%) | **0.018** | **5.18 (1.15-23.26, 0.032)** |
|  | Second | 5 (1.9%) | 3 (1.9%) | 2 (1.9%) | 1.000 | 1.03 (0.17-6.27, 0.97) |
| Joint pain | First | 13 (4.9%) | 13 (8.3%) | 0 | **0.002** | >100 (0 to uncertain, >0.99) ‡ |
|  | Second | 4 (1.5%) | 4 (2.6%) | 0 | 0.095 | >100 (0 to uncertain, >0.99) ‡ |
| Local stiffness | First | 11 (4.2%) | 7 (4.5%) | 4 (3.7%) | 1.000 | 1.21 (0.34-4.24, 0.77) |
|  | Second | 4 (1.5%) | 2 (1.3%) | 2 (1.9%) | 1.000 | 0.68 (0.09-4.92, 0.70) |
| Local swelling | First | 8 (3.0%) | 8 (5.1%) | 0 | **0.023** | >100 (0 to uncertain, >0.99) ‡ |
|  | Second | 2 (0.8%) | 0 | 2 (1.9%) | 0.165 | 0 (0 to uncertain, >0.99) § |
| Local warming | First | 6 (2.3%) | 4 (2.6%) | 2 (1.9%) | 1.000 | 1.38 (0.25-7.68, 0.71) |
|  | Second | 6 (2.3%) | 3 (1.9%) | 3 (2.8%) | 0.690 | 0.68 (0.13-3.43, 0.64) |
| Other symptoms | First | 6 (2.3%) | 4 (2.6%) | 2 (1.9%) | 1.000 | 1.38 (0.25-7.68, 0.71) |
|  | Second | 2 (0.8%) | 0 | 2 (1.9%) | 0.165 | 0 (0 to uncertain, >0.99) § |
| Rhinorrhea | First | 4 (1.5%) | 3 (1.9%) | 1 (0.9%) | 0.648 | 2.08 (0.21-20.25, 0.53) |
|  | Second | 0 | 0 | 0 | --- | --- |
| Local redness | First | 3 (1.1%) | 3 (1.9%) | 0 | 0.273 | >100 (0 to uncertain, >0.99) ‡ |
|  | Second | 0 | 0 | 0 | --- | --- |
| Rash | First | 2 (0.8%) | 2 (1.3%) | 0 | 0.516 | >100 (0 to uncertain, >0.99) ‡ |
|  | Second | 1 (0.4%) | 0 | 1 (0.9%) | 0.407 | 0 (0 to uncertain, >0.99) § |
| Hypotension | First | 2 (0.8%) | 1 (0.6%) | 1 (0.9%) | 1.000 | 0.68 (0.04-11.5, 0.79) |
|  | Second | 0 | 0 | 0 | --- | --- |
| Eye pain | First | 2 (0.8%) | 2 (1.3%) | 0 | 0.516 | >100 (0 to uncertain, >0.99) ‡ |
|  | Second | 0 | 0 | 0 | --- | --- |
| Depression | First | 2 (0.8%) | 2 (1.3%) | 0 | 0.516 | >100 (0 to uncertain, >0.99) ‡ |
|  | Second | 0 | 0 | 0 | --- | --- |
| Palpitation | First | 2 (0.8%) | 2 (1.3%) | 0 | 0.516 | >100 (0 to uncertain, >0.99) ‡ |
|  | Second | 0 | 0 | 0 | --- | --- |
| Itching | First | 2 (0.8%) | 2 (1.3%) | 0 | 0.516 | >100 (0 to uncertain, >0.99) ‡ |
|  | Second | 0 | 0 | 0 | --- | --- |
| Aphthous stomatitis | First | 1 (0.4%) | 0 | 1 (0.9%) | 0.407 | 0 (0 to uncertain, >0.99) § |
|  | Second | 1 (0.4%) | 0 | 1 (0.9%) | 0.407 | 0 (0 to uncertain, >0.99) § |
| Tingling of the mouth/tongue/lips | First | 1 (0.4%) | 1 (0.6%) | 0 | 1.000 | >100 (0 to uncertain, >0.99) ‡ |
|  | Second | 0 | 0 | 0 | --- | --- |
| Urticaria | First | 1 (0.4%) | 1 (0.6%) | 0 | 1.000 | >100 (0 to uncertain, >0.99) ‡ |
|  | Second | 0 | 0 | 0 | --- | --- |
| Xerostomia | First | 1 (0.4%) | 1 (0.6%) | 0 | 1.000 | >100 (0 to uncertain, >0.99) ‡ |
|  | Second | 0 | 0 | 0 | --- | --- |
| Petechiae | First | 0 | 0 | 0 | --- | --- |
|  | Second | 0 | 0 | 0 | --- | --- |
| Other skin symptoms | First | 0 | 0 | 0 | --- | --- |
|  | Second | 0 | 0 | 0 | --- | --- |
| Eye redness | First | 0 | 0 | 0 | --- | --- |
|  | Second | 0 | 0 | 0 | --- | --- |
| Delirium | First | 0 | 0 | 0 | --- | --- |
|  | Second | 0 | 0 | 0 | --- | --- |
| Impaired consciousness | First | 0 | 0 | 0 | --- | --- |
|  | Second | 0 | 0 | 0 | --- | --- |
| Lymphadenopathy | First | 0 | 0 | 0 | --- | --- |
|  | Second | 0 | 0 | 0 | --- | --- |
| Bleeding gums | First | 0 | 0 | 0 | --- | --- |
|  | Second | 0 | 0 | 0 | --- | --- |
| Swelling of the mouth/tongue/cheek | First | 0 | 0 | 0 | --- | --- |
|  | Second | 0 | 0 | 0 | --- | --- |
| Oral blister | First | 0 | 0 | 0 | --- | --- |
|  | Second | 0 | 0 | 0 | --- | --- |
| Halitosis | First | 0 | 0 | 0 | --- | --- |
|  | Second | 0 | 0 | 0 | --- | --- |
| Mouth ulcers | First | 0 | 0 | 0 | --- | --- |
|  | Second | 0 | 0 | 0 | --- | --- |
| Oral white/red plaque | First | 0 | 0 | 0 | --- | --- |
|  | Second | 0 | 0 | 0 | --- | --- |
| Taste disturbance | First | 0 | 0 | 0 | --- | --- |
|  | Second | 0 | 0 | 0 | --- | --- |

† Odds ratio (95% confidence interval). The Sinopharm group was designated as the reference group, serving as the baseline for comparison.

‡ This extremely high odds ratio with the uncertainty in the confidence interval is likely due to the rarity of events in the reference group. Despite the high odds ratio, the non-significant p-value suggests that there is no statistically significant difference in the odds of the adverse event between the Sinopharm and Oxford–AstraZeneca groups.

§ The zero odds ratio with a wide or uncertain confidence interval is likely due to the lack of events in the comparison group.
