# Supplementary material for: Self‐Reported Adverse Events Following COVID‐19 Vaccination Among Medical Sciences Students After a Symptomatology Training Program: A Cross‐Sectional Study
Source: Health Sci Rep. 2025 Mar 2;8(3):e70492. doi: 10.1002/hsr2.70492 (PMC11872685; doi:10.1002/hsr2.70492)
Supplement: Supplementary file 2 — Supporting information. [file HSR2-8-e70492-s004.docx]

**Supplemental Table S2.** **Central tendencies and dispersion indicators of adverse events in participants reporting adverse events following first dose administration**

| First dose | | Total | | | Vaccines | | | | | | |
| --- | --- | --- | --- | --- | --- | --- | --- | --- | --- | --- | --- |
|  |  |  |  |  | Oxford–AstraZeneca | | | Sinopharm | | | p-value† |
| Adverse events | Time (Hours) | Mean (SD) | Min-Max | Median (IQR) | Mean (SD) | Min-Max | Median (IQR) | Mean (SD) | Min-Max | Median (IQR) |  |
| Total | Initiation | 11.1 (13.8) | 1-168 | 10 (7-12) | 10.6 (5.2) | 1-48 | 10 (8-12) | 14.2 (34.9) | 1-168 | 4.5 (3-8) | **<0.001** |
|  | Duration | 32.2 (48.9) | 1->1000 | 24 (12-48) | 32.7 (50.7) | 1->1000 | 24 (12-48) | 29.2 (37.2) | 1-240 | 18 (7-48) | **0.003** |
| Injection site pain | Initiation | 10.1 (16.8) | 1-168 | 8 (4-12) | 10.4 (7.6) | 1-48 | 10 (6-12) | 9.6 (27) | 1-168 | 4 (1-7.3) | **<0.001** |
|  | Duration | 38.6 (40.3) | 1-240 | 24 (12-48) | 42.7 (39) | 1-168 | 24 (24-48) | 31.3 (42.2) | 1-240 | 24 (8-48) | **0.02** |
| Fever | Initiation | 11.5 (15.6) | 3-168 | 10 (8-12) | 10.3 (3.8) | 3-24 | 10 (8-12) | 32.9 (66) | 3-168 | 7 (3-49) | 0.100 |
|  | Duration | 23.2 (17.9) | 1-72 | 19 (8-36) | 23.8 (18) | 1-72 | 22.5 (8.8-37) | 13.3 (12.9) | 1-36 | 10.5 (3-22.5) | 0.14 |
| Body/muscle pain | Initiation | 10 (4.27) | 3-24 | 10 (8-12) | 10.2 (4.2) | 3-24 | 10 (8-12) | 5 (2.8) | 3-7 | 5 (3-?) | **0.04** |
|  | Duration | 33 (26.3) | 2-168 | 30 (12-48) | 34 (26.4) | 2-168 | 30 (14-48) | 9.5 (3.5) | 7-12 | 9.5 (7-?) | 0.08 |
| Chills | Initiation | 9.6 (3.2) | 3-20 | 10 (7-12) | 9.4 (3) | 3-15 | 10 (7-12) | 20 (0) | 20-20 | 20 (20-20) | 0.08 |
|  | Duration | 20.4 (16.8) | 1-72 | 17 (6-24) | 20.6 (16.7) | 1-72 | 17.5 (6-25.5) | 2 (0) | 2-2 | 2 (2-2) | 0.13 |
| Headache | Initiation | 12.2 (19.8) | 1-168 | 9 (7-12) | 10.5 (4.43) | 1-24 | 10 (8-12) | 21.7 (51.5) | 1-168 | 6.5 (2.8-8.3) | **0.01** |
|  | Duration | 29.9 (23.3) | 2-120 | 24 (12-48) | 31 (23.4) | 2-120 | 24 (12-48) | 24.8 (23.2) | 3-72 | 13 (8.3-48) | 0.38 |
| Feeling unwell | Initiation | 10.1 (5) | 2-24 | 10 (6-12) | 11 (4.3) | 5-24 | 10 (8-12) | 6.2 (6.5) | 2-24 | 4 (3-6.5) | **<0.001** |
|  | Duration | 34.4 (21.8) | 3-120 | 24 (22.5-48) | 34.8 (18) | 3-72 | 36 (24-48) | 33 (33.1) | 3-120 | 24 (15.8-39) | 0.25 |
| Fatigue | Initiation | 9.5 (4.2) | 3-24 | 8 (6-12) | 10.2 (4) | 5-24 | 10 (8-12) | 5.5 (3.1) | 3-12 | 4 (3.3-7.5) | **0.001** |
|  | Duration | 34.8 (25.3) | 3-120 | 30 (12-48) | 36 (25.4) | 3-120 | 30 (13.5-48) | 29 (25.6) | 3-72 | 24 (6.3-48) | 0.41 |
| Sweating | Initiation | 10.3 (4) | 5-20 | 10 (8-12) | 9.8 (3.5) | 5-20 | 9 (7.8-12) | 20 (0) | 20-20 | 20 (20-20) | 0.11 |
|  | Duration | 29.4 (15.5) | 2-72 | 24 (21-36) | 31 (14.5) | 18-72 | 24 (23.3-39) | 2 (0) | 2-2 | 2 (2-2) | 0.09 |
| Nausea | Initiation | 12.6 (4.9) | 6-24 | 12 (8-14) | 12.6 (5.1) | 6-24 | 12 (8-15) | 12 (0) | 12-12 | 12 (12-12) | >0.99 |
|  | Duration | 24.5 (21.4) | 1-72 | 24 (8.5-42) | 26.5 (21) | 1-72 | 24 (12-45) | 1 (0) | 1-1 | 1 (1-1) | 0.10 |
| Dizziness | Initiation | 13 (5.3) | 6-24 | 12 (17.25-8) | 12.7 (4.5) | 6-20 | 12 (8-15.8) | 16 (11.3) | 8-24 | 16 (8-?) | 0.63 |
|  | Duration | 39.8 (36.7) | 3-120 | 24 (10-72) | 40 (37) | 3-120 | 24 (11-60) | 38 (48) | 4-72 | 38 (4-?) | 0.91 |
| Joint pain | Initiation | 9.6 (2.4) | 5-13 | 9 (8-12) | 9.6 (2.4) | 5-13 | 9 (8-12) | -- | -- | -- | -- |
|  | Duration | 161 (370) | 2->1000 | 24 (3-48) | 161 (370) | 2->1000 | 24 (3-48) | -- | -- | -- | -- |
| Local stiffness | Initiation | 9.2 (6.3) | 1-18 | 12 (1-12) | 12.4 (3.6) | 8-18 | 12 (10-15) | 1 (0) | 1-1 | 1 (1-1) | **0.04** |
|  | Duration | 30 (15.5) | 12-48 | 30 (45-15) | 36 (12) | 24-48 | 36 (24-?) | 12 (0) | 12-12 | 12 (12-12) | 0.18 |
| Local swelling | Initiation | 12.1 (6.1) | 5-24 | 11 (16.5-8) | 12.1 (6) | 5-24 | 11 (8-16.5) | -- | -- | -- | -- |
|  | Duration | 48 (15.2) | 24-72 | 48 (42-57) | 48 (15) | 24-72 | 48 (42-54) | -- | -- | -- | -- |
| Local warming | Initiation | 8 (10.4) | 1-20 | 3 (1-?) | 20 (0) | 20-20 | 20 (20-20) | 2 (1.4) | 1-3 | 2 (1-?) | 0.22 |
|  | Duration | 4 (0) | 4-4 | 4 (4-?) | 6 (0) | 6-6 | 6 (6-6) | 4 (0) | 4-4 | 4 (4-4) | 0.22 |
| Other symptoms | Initiation | -- | -- | -- | -- | -- | -- | -- | -- | -- | -- |
|  | Duration | -- | -- | -- | -- | -- | -- | -- | -- | -- | -- |
| Rhinorrhea | Initiation | 63 (91) | 7-168 | 15 (7-?) | 11 (5.7) | 7-15 | 11 (7-?) | 168 (0) | 168-168 | 168 (168-168) | 0.22 |
|  | Duration | 24 (0) | 24-24 | 24 (24-?) | 24 (0) | 24-24 | 24 (24-24) | 24 (0) | 24-24 | 24 (24-24) | >0.99 |
| Local redness | Initiation | 8 (2) | 6-10 | 8 (6-?) | 8 (2) | 6-10 | 8 (6-?) | -- | -- | -- | -- |
|  | Duration | 36 (17) | 24-48 | 36 (24-?) | 36 (17) | 24-48 | 36 (24-?) | -- | -- | -- | -- |
| Rash | Initiation | 48 (0) | 48-48 | 48 (48-48) | 48 (0) | 48-48 | 48 (48-48) | -- | -- | -- | -- |
|  | Duration | 72 (0) | 72-72 | 72 (72-72) | 72 (0) | 72-72 | 72 (72-72) | -- | -- | -- | -- |
| Hypotension | Initiation | 21 (4.24) | 18-24 | 21 (18-?) | 18 (0) | 18-18 | 18 (18-18) | 24 (0) | 24-24 | 24 (24-24) | 0.32 |
|  | Duration | 38.5 (47.4) | 5-72 | 38.5 (5-?) | 5 (0) | 5-5 | 5 (5-5) | 72 (0) | 72-72 | 72 (72-72) | 0.32 |
| Eye pain | Initiation | 11 (1.41) | 10-12 | 11 (10-?) | 11 (1.4) | 10-12 | 11 (10-?) | -- | -- | -- | -- |
|  | Duration | 9 (0) | 9-9 | 9 (9-9) | 9 (0) | 9-9 | 9 (9-9) | -- | -- | -- | -- |
| Depression | Initiation | 48 (0) | 48-48 | 48 (48-48) | 48 (0) | 48-48 | 48 (48-48) | -- | -- | -- | -- |
|  | Duration | 120 (0) | 120-120 | 120 (120-120) | 120 (0) | 120-120 | 120 (120-120) | -- | -- | -- | -- |
| Palpitation | Initiation | 20 (0) | 20-20 | 20 (20-20) | 20 (0) | 20-20 | 20 (20-20) | -- | -- | -- | -- |
|  | Duration | 10 (0) | 10-10 | 10 (10-10) | 10 (0) | 10-10 | 10 (10-10) | -- | -- | -- | -- |
| Itching | Initiation | 28 (28.3) | 8-48 | 28 (8-?) | 28 (28.3) | 8-48 | 28 (8-?) | -- | -- | -- | -- |
|  | Duration | 24 (0) | 24-24 | 24 214-24) | 24 (0) | 24-24 | 24 (24-24) | -- | -- | -- | -- |
| Aphthous stomatitis | Initiation | 48 (0) | 48-48 | 48 (48-48) | -- | -- | -- | 48 (0) | 48-48 | 48 (48-48) | -- |
|  | Duration | 168 (0) | 168-168 | 168 (168-168) | -- | -- | -- | 168 (0) | 168-168 | 168 (168-168) | -- |
| Tingling of the mouth/tongue/lips | Initiation | 1 (0) | 1-1 | 1 (1-1) | 1 (0) | 1-1 | 1 (1-1) | -- | -- | -- | -- |
|  | Duration | 24 (0) | 24-24 | 24 (24-24) | 24 (0) | 24-24 | 24 (24-24) | -- | -- | -- | -- |
| Urticaria | Initiation | 24 (0) | 24-24 | 24 (24-24) | 24 (0) | 24-24 | 24 (24-24) | -- | -- | -- | -- |
|  | Duration | 48 (0) | 48-48 | 48 (48-48) | 48 (0) | 48-48 | 48 (48-48) | -- | -- | -- | -- |
| Xerostomia | Initiation | 24 (0) | 24-24 | 24 (24-24) | 24 (0) | 24-24 | 24 (24-24) | -- | -- | -- | -- |
|  | Duration | 36 (0) | 36-36 | 36 (36-36) | 36 (0) | 36-36 | 36 (36-36) | -- | -- | -- | -- |
| Petechiae | Initiation | -- | -- | -- | -- | -- | -- | -- | -- | -- | -- |
|  | Duration | -- | -- | -- | -- | -- | -- | -- | -- | -- | -- |
| Other skin symptoms | Initiation | -- | -- | -- | -- | -- | -- | -- | -- | -- | -- |
|  | Duration | -- | -- | -- | -- | -- | -- | -- | -- | -- | -- |
| Eye redness | Initiation | -- | -- | -- | -- | -- | -- | -- | -- | -- | -- |
|  | Duration | -- | -- | -- | -- | -- | -- | -- | -- | -- | -- |
| Delirium | Initiation | -- | -- | -- | -- | -- | -- | -- | -- | -- | -- |
|  | Duration | -- | -- | -- | -- | -- | -- | -- | -- | -- | -- |
| Impaired consciousness | Initiation | -- | -- | -- | -- | -- | -- | -- | -- | -- | -- |
|  | Duration | -- | -- | -- | -- | -- | -- | -- | -- | -- | -- |
| Lymphadenopathy | Initiation | -- | -- | -- | -- | -- | -- | -- | -- | -- | -- |
|  | Duration | -- | -- | -- | -- | -- | -- | -- | -- | -- | -- |
| Bleeding gums | Initiation | -- | -- | -- | -- | -- | -- | -- | -- | -- | -- |
|  | Duration | -- | -- | -- | -- | -- | -- | -- | -- | -- | -- |
| Swelling of the mouth/tongue/cheek | Initiation | -- | -- | -- | -- | -- | -- | -- | -- | -- | -- |
|  | Duration | -- | -- | -- | -- | -- | -- | -- | -- | -- | -- |
| Oral blister | Initiation | -- | -- | -- | -- | -- | -- | -- | -- | -- | -- |
|  | Duration | -- | -- | -- | -- | -- | -- | -- | -- | -- | -- |
| Halitosis | Initiation | -- | -- | -- | -- | -- | -- | -- | -- | -- | -- |
|  | Duration | -- | -- | -- | -- | -- | -- | -- | -- | -- | -- |
| Mouth ulcers | Initiation | -- | -- | -- | -- | -- | -- | -- | -- | -- | -- |
|  | Duration | -- | -- | -- | -- | -- | -- | -- | -- | -- | -- |
| Oral white/red plaque | Initiation | -- | -- | -- | -- | -- | -- | -- | -- | -- | -- |
|  | Duration | -- | -- | -- | -- | -- | -- | -- | -- | -- | -- |
| Taste disturbance | Initiation | -- | -- | -- | -- | -- | -- | -- | -- | -- | -- |
|  | Duration | -- | -- | -- | -- | -- | -- | -- | -- | -- | -- |

†Mann-Whitney U test.

**--:** Zero frequency.

**?:** Low frequency resulted in no reports (primarily observed in variables with frequencies of 2 or 3).

**>1000:** The duration was prolonged; the symptom persisted at the time of assessment.
