# Supplementary material for: Self‐Reported Adverse Events Following COVID‐19 Vaccination Among Medical Sciences Students After a Symptomatology Training Program: A Cross‐Sectional Study
Source: Health Sci Rep. 2025 Mar 2;8(3):e70492. doi: 10.1002/hsr2.70492 (PMC11872685; doi:10.1002/hsr2.70492)
Supplement: Supplementary file 3 — Supporting information. [file HSR2-8-e70492-s002.docx]

**Supplemental Table S3.** **Central tendencies and dispersion indicators of adverse events in participants reporting adverse events following second dose administration**

| Second dose | | Total | | | Vaccines | | | | | |  |
| --- | --- | --- | --- | --- | --- | --- | --- | --- | --- | --- | --- |
|  |  |  |  |  | Oxford–AstraZeneca | | | Sinopharm | | | p-value† |
| Adverse events | Time (Hours) | Mean (SD) | Min-Max | Median (IQR) | Mean (SD) | Min-Max | Median (IQR) | Mean (SD) | Min-Max | Median (IQR) |  |
| Total | Initiation | 9.9 (21.7) | 1-336 | 8 (4-12) | 10 (5.2) | 1-24 | 10 (6-12) | 9.6 (37.9) | 1-336 | 3 (2-6) | **˂0.001** |
|  | Duration | 27.7 (29.4) | 1-168 | 24 (10-36) | 29.1 (30.2) | 1-168 | 24 (10-36) | 25 (27.7) | 1-168 | 15 (8-24) | 0.25 |
| Injection site pain | Initiation | 6.5 (6) | 1-24 | 4 (2-10) | 8.7 (5.6) | 1-24 | 8 (3.8-12) | 3.8 (5.3) | 1-24 | 2 (1-4) | **˂0.001** |
|  | Duration | 31.2 (29) | 1-168 | 24 (12-48) | 35.5 (33.4) | 1-168 | 24 (12-48) | 26 (22) | 2-72 | 24 (12-36) | 0.17 |
| Fever | Initiation | 10 (4.6) | 2-24 | 9 (6.5-12) | 10.9 (4.5) | 5-24 | 10 (8-14.3) | 6.5 (3.9) | 2-12 | 6 (2.8-10.5) | **0.04** |
|  | Duration | 19 (20.6) | 1-96 | 12 (6-24) | 20.8 (22.7) | 1-96 | 14 (5.5-24) | 12.9 (6.8) | 1-24 | 12 (12-15) | 0.70 |
| Body/muscle pain | Initiation | 9.8 (5.7) | 2-24 | 8 (6-12) | 9.6 (5.1) | 2-24 | 8 (6-12) | 11 (9.6) | 2-24 | 9 (3-21) | 0.97 |
|  | Duration | 34.4 (39) | 4-168 | 24 (12-36) | 27.2 (24.7) | 4-96 | 24 (12-36) | 77.8 (79) | 8-168 | 67.5 (9.8-156) | 0.39 |
| Chills | Initiation | 12.8 (5.5) | 4-24 | 12 (8-16.5) | 12.8 (5.5) | 4-24 | 12 (8-16.5) | -- | -- | -- | -- |
|  | Duration | 19.3 (28) | 1-96 | 9 (1.3-30) | 19.3 (28) | 1-96 | 9 (1.3-30) | -- | -- | -- | -- |
| Headache | Initiation | 9.6 (6.1) | 2-24 | 8 (5-12.5) | 11.8 (5.7) | 4-24 | 11 (7.3-15.3) | 7 (5.8) | 2-24 | 6 (3.3-7.8) | **0.01** |
|  | Duration | 28.7 (37.9) | 1-168 | 14 (10-24) | 39.5 (49) | 4-168 | 21 (10.5-60) | 16.8 (14.7) | 1-48 | 12 (6-24) | 0.23 |
| Feeling unwell | Initiation | 8.7 (4.3) | 3-18 | 8 (5.3-12) | 10 (4) | 4-18 | 10 (6-12) | 4.6 (2) | 3-8 | 4 (3-6.5) | **0.007** |
|  | Duration | 21.4 (20.4) | 6-96 | 16 (12-24) | 23.5 (23.4) | 6-96 | 17 (11.5-24) | 15.6 (5.4) | 12-24 | 12 (12-21) | 0.74 |
| Fatigue | Initiation | 9.7 (6.4) | 4-24 | 7 (4.8-13) | 10.9 (6.7) | 4-24 | 10 (5.3-15) | 5 (1.4) | 4-6 | 5 (4-?) | 0.19 |
|  | Duration | 22.8 (26.7) | 4-96 | 12 (10-24) | 25 (29.3) | 10-96 | 12 (10.5-24) | 14 (14.1) | 4-24 | 14 (4-?) | 0.59 |
| Sweating | Initiation | 8.7 (1) | 8-10 | 8 (8-10) | 8.7 (1) | 8-10 | 8 (8-10) | -- | -- | -- | -- |
|  | Duration | 36.8 (33) | 5-96 | 30 (10.3-60) | 36.8 (33) | 5-96 | 30 (10.3-60) | -- | -- | -- | -- |
| Nausea | Initiation | 8.3 (3.9) | 3-12 | 9 (4.3-11.5) | 8.3 (3.9) | 3-12 | 9 (4.3-11.5) | -- | -- | -- | -- |
|  | Duration | 45 (34.5) | 24-96 | 30 (24-81) | 45 (34.5) | 24-96 | 30 (24-81) | -- | -- | -- | -- |
| Dizziness | Initiation | 7.8 (7.6) | 2-20 | 5 (2-15) | 11.7 (7.7) | 5-20 | 10 (5-?) | 2 (0) | 2-2 | 2 (2-2) | 0.08 |
|  | Duration | 15.8 (5.7) | 12-24 | 13.5 (12-21.8) | 18 (8.5) | 12-24 | 18 (12-?) | 13.5 (2) | 12-15 | 13.5 (12-?) | 0.68 |
| Joint pain | Initiation | 8.8 (4.3) | 3-12 | 10 (4.3-12) | 8.8 (4.3) | 3-12 | 10 (4.3-12) | -- | -- | -- | -- |
|  | Duration | 21 (11.5) | 12-36 | 18 (12-33) | 21 (11.5) | 12-36 | 18 (12-33) | -- | -- | -- | -- |
| Local stiffness | Initiation | 5 (3.6) | 1-8 | 6 (1-?) | 8 (0) | 8-8 | 8 (8-8) | 3.5 (3.6) | 1-6 | 3.5 (1-?) | 0.22 |
|  | Duration | 32 (7) | 24-36 | 36 (24-?) | 36 (0) | 36-36 | 36 (36-36) | 30 (8.5) | 24-36 | 30 (24-?) | 0.48 |
| Local swelling | Initiation | 2 (1.4) | 1-3 | 2 (1-?) | -- | -- | -- | 2 (1.4) | 1-3 | 2 (1-?) | -- |
|  | Duration | 3.5 (3.6) | 1-6 | 3.5 (1-?) | -- | -- | -- | 3.5 (3.6) | 1-6 | 3.5 (1-?) | -- |
| Local warming | Initiation | 5.8 (6.9) | 1-16 | 3 (1.5-12.8) | 16 (0) | 16-16 | 16 (16-16) | 2.3 (1.2) | 1-3 | 3 (1-?) | 0.16 |
|  | Duration | 11 (11.4) | 3-24 | 6 (3-8) | 24 (0) | 24-24 | 24 (24-24) | 11 (11.4) | 3-24 | 6 (3-?) | 0.20 |
| Other symptoms | Initiation | -- | -- | -- | -- | -- | -- | -- | -- | -- | -- |
|  | Duration | -- | -- | -- | -- | -- | -- | -- | -- | -- | -- |
| Rhinorrhea | Initiation | -- | -- | -- | -- | -- | -- | -- | -- | -- | -- |
|  | Duration | -- | -- | -- | -- | -- | -- | -- | -- | -- | -- |
| Local redness | Initiation | -- | -- | -- | -- | -- | -- | -- | -- | -- | -- |
|  | Duration | -- | -- | -- | -- | -- | -- | -- | -- | -- | -- |
| Rash | Initiation | 336 (0) | 336-336 | 336 (336-336) | -- | -- | -- | 336 (0) | 336-336 | 336 (336-336) | -- |
|  | Duration | 72 (0) | 72-72 | 72 (72-72) | -- | -- | -- | 72 (0) | 72-72 | 72 (72-72) | -- |
| Hypotension | Initiation | -- | -- | -- | -- | -- | -- | -- | -- | -- | -- |
|  | Duration | -- | -- | -- | -- | -- | -- | -- | -- | -- | -- |
| Eye pain | Initiation | -- | -- | -- | -- | -- | -- | -- | -- | -- | -- |
|  | Duration | -- | -- | -- | -- | -- | -- | -- | -- | -- | -- |
| Depression | Initiation | -- | -- | -- | -- | -- | -- | -- | -- | -- | -- |
|  | Duration | -- | -- | -- | -- | -- | -- | -- | -- | -- | -- |
| Palpitation | Initiation | -- | -- | -- | -- | -- | -- | -- | -- | -- | -- |
|  | Duration | -- | -- | -- | -- | -- | -- | -- | -- | -- | -- |
| Itching | Initiation | -- | -- | -- | -- | -- | -- | -- | -- | -- | -- |
|  | Duration | -- | -- | -- | -- | -- | -- | -- | -- | -- | -- |
| Aphthous stomatitis | Initiation | 48 (0) | 48-48 | 48 (48-48) | -- | -- | -- | 48 (0) | 48-48 | 48 (48-48) | -- |
|  | Duration | 72 (0) | 72-72 | 72 (72-72) | -- | -- | -- | 72 (0) | 72-72 | 72 (72-72) | -- |
| Tingling of the mouth/tongue/lips | Initiation | -- | -- | -- | -- | -- | -- | -- | -- | -- | -- |
|  | Duration | -- | -- | -- | -- | -- | -- | -- | -- | -- | -- |
| Urticaria | Initiation | -- | -- | -- | -- | -- | -- | -- | -- | -- | -- |
|  | Duration | -- | -- | -- | -- | -- | -- | -- | -- | -- | -- |
| Xerostomia | Initiation | -- | -- | -- | -- | -- | -- | -- | -- | -- | -- |
|  | Duration | -- | -- | -- | -- | -- | -- | -- | -- | -- | -- |
| Petechiae | Initiation | -- | -- | -- | -- | -- | -- | -- | -- | -- | -- |
|  | Duration | -- | -- | -- | -- | -- | -- | -- | -- | -- | -- |
| Other skin symptoms | Initiation | -- | -- | -- | -- | -- | -- | -- | -- | -- | -- |
|  | Duration | -- | -- | -- | -- | -- | -- | -- | -- | -- | -- |
| Eye redness | Initiation | -- | -- | -- | -- | -- | -- | -- | -- | -- | -- |
|  | Duration | -- | -- | -- | -- | -- | -- | -- | -- | -- | -- |
| Delirium | Initiation | -- | -- | -- | -- | -- | -- | -- | -- | -- | -- |
|  | Duration | -- | -- | -- | -- | -- | -- | -- | -- | -- | -- |
| Impaired consciousness | Initiation | -- | -- | -- | -- | -- | -- | -- | -- | -- | -- |
|  | Duration | -- | -- | -- | -- | -- | -- | -- | -- | -- | -- |
| Lymphadenopathy | Initiation | -- | -- | -- | -- | -- | -- | -- | -- | -- | -- |
|  | Duration | -- | -- | -- | -- | -- | -- | -- | -- | -- | -- |
| Bleeding gums | Initiation | -- | -- | -- | -- | -- | -- | -- | -- | -- | -- |
|  | Duration | -- | -- | -- | -- | -- | -- | -- | -- | -- | -- |
| Swelling of the mouth/tongue/cheek | Initiation | -- | -- | -- | -- | -- | -- | -- | -- | -- | -- |
|  | Duration | -- | -- | -- | -- | -- | -- | -- | -- | -- | -- |
| Oral blister | Initiation | -- | -- | -- | -- | -- | -- | -- | -- | -- | -- |
|  | Duration | -- | -- | -- | -- | -- | -- | -- | -- | -- | -- |
| Halitosis | Initiation | -- | -- | -- | -- | -- | -- | -- | -- | -- | -- |
|  | Duration | -- | -- | -- | -- | -- | -- | -- | -- | -- | -- |
| Mouth ulcers | Initiation | -- | -- | -- | -- | -- | -- | -- | -- | -- | -- |
|  | Duration | -- | -- | -- | -- | -- | -- | -- | -- | -- | -- |
| Oral white/red plaque | Initiation | -- | -- | -- | -- | -- | -- | -- | -- | -- | -- |
|  | Duration | -- | -- | -- | -- | -- | -- | -- | -- | -- | -- |
| Taste disturbance | Initiation | -- | -- | -- | -- | -- | -- | -- | -- | -- | -- |
|  | Duration | -- | -- | -- | -- | -- | -- | -- | -- | -- | -- |

†Mann-Whitney U test.

**--:** Zero frequency.

**?:** Low frequency resulted in no reports (primarily observed in variables with frequencies of 2 or 3).

**>1000:** The duration was prolonged; the symptom persisted at the time of assessment.
