# Supplementary material for: Self‐Reported Adverse Events Following COVID‐19 Vaccination Among Medical Sciences Students After a Symptomatology Training Program: A Cross‐Sectional Study
Source: Health Sci Rep. 2025 Mar 2;8(3):e70492. doi: 10.1002/hsr2.70492 (PMC11872685; doi:10.1002/hsr2.70492)
Supplement: Supplementary file 4 — Supporting information. [file HSR2-8-e70492-s003.docx]

**Supplemental Table S4: Distribution of medications used to alleviate adverse events**

|  | **Dose** | **Total** | **Oxford–AstraZeneca** | **Sinopharm** |
| --- | --- | --- | --- | --- |
| **Medication use to relieve symptoms, N (%)** | First | 158 | 130 | 28 |
|  | Second | 69 | 57 | 12 |
| Acetaminophen | First | 112 (70.89%) | 85 (65.38%) | 27 (96.43%) |
|  | Second | 55 (79.72%) | 44 (77.19%) | 11 (91.7%) |
| Acetaminophen + NSAID | First | 28 (17.72%) | 28 (21.54%) | 0 |
|  | Second | 5 (7.24%) | 4 (7.03%) | 1 (8.3%) |
| Acetaminophen + NSAID + other drugs | First | 5 (3.16%) | 4 (3.08%) | 1 (3.57%) |
|  | Second | 1 (1.45%) | 1 (1.75%) | 0 |
| Acetaminophen + Other drugs | First | 4 (2.53%) | 4 (3.08%) | 0 |
|  | Second | 0 | 0 | 0 |
| NSAID | First | 8 (5.06%) | 8 (6.15%) | 0 |
|  | Second | 8 (11.59%) | 8 (14.03%) | 0 |
| NSAID + Other drugs | First | 1 (0.64%) | 1 (0.77%) | 0 |
|  | Second | 0 | 0 | 0 |
| Other drugs | First | 0 | 0 | 0 |
|  | Second | 0 | 0 | 0 |

NSAID: nonsteroidal anti-inflammatory drug
